# Retrospect

**Published:** 1887-12

**Authors:** 


### RETROSPECT.

My summer-day friends are gone,
  They came with the robins, and sped
When the robins forsook the velvety lawn,
  And the maples overhead.

We roved where the violets grew,
  In groves and meadows rare,
Whilst over our northern hills there drew,
  A soft delicious air.

In many a leafy nook,
  And on the mountains bare,
We saw in nature's holiday book,
  That earth is wond'rous fair.

Where tamaracks veiled the sun,
  We carved our names in a line,
Ah ! why should I always dream of the one
  Whose name is nearest mine ?

Shall we ever meet again,
  Those summer-day friends and I ?
I ask of the wandering winds, but then
  I only hear a sigh !

